# Response to Letter to the Editor

**DOI:** 10.1055/s-0038-1673375

**Published:** 2018-12-10

**Authors:** Christian Bonde

**Affiliations:** 1 Department of Neurosurgery, Odense University Hospital, Odense, Denmark

No Value of Routine Brain Computed Tomography 6 Weeks after Evacuation of Chronic Subdural Hematoma

Thanks for forwarding the “letter to the editor,” giving me an opportunity to answer

1. It is a comment, and we fully agree.
2. We agree that Glasgow coma scale is a poor indicator, when it comes to chronic subdural hematoma. However, the medical records were insufficient to use a better grading system.
3. To clarify this, there were 202 patients but 4 were lost to follow-up.
4. The statical methods could be described but was not due to limitation in publication size.
5. The adjectives were used in order to have some variation in language.
6. We do not think that this issue should be discussed in the present article.

